# Campaign-style enforcement and corporate environmental governance: evidence from China’s central environmental inspection

**DOI:** 10.3389/fpubh.2025.1688719

**Published:** 2025-11-05

**Authors:** Yukai Dong, Junrong Huang, Yuhong Li

**Affiliations:** ^1^School of Public Affairs, Xiamen University, Xiamen, China; ^2^School of Accountancy, Xinjiang University of Finance and Economics, Urumqi, China

**Keywords:** campaign-style enforcement, corporate environmental governance, central environmental inspection, government-business relations, corporate environmental investment

## Abstract

Campaign-style enforcement is a crucial approach to bridging enforcement gaps and improving environmental quality. Existing literature has largely focused on its impacts on environmental performance, government actions, and public response, while relatively neglecting its effects on enterprises. Using a staggered difference-in-differences (DID) approach and data from China’s Central Environmental Inspection (CEI), this study examines the impact and mechanisms of campaign-style enforcement on corporate environmental governance, as well as the moderating role of government–business relations. Results show that campaign-style enforcement can lead heavily polluting enterprises to increase their environmental investments, though this effect is weakened when government–business relations are close. Furthermore, under the pressure of the CEI, local governments primarily employ punitive measures rather than financial incentives to prompt these enterprises to increase their environmental investments. Heterogeneity analysis reveals that campaign-style enforcement has a more pronounced effect on small firms and firms located in eastern regions. These results highlight regional differences in enforcement effectiveness and enrich understanding of how campaign-style environmental enforcement shapes corporate behavior, offering valuable insights for future CEI policies.

## 1 Introduction

Since the reform and opening up, China has achieved remarkable economic miracles, but it also faces challenges related to ecological degradation and environmental pollution. How to address and manage the worsening ecological environment and the resulting political, economic, and social issues has become a key policy concern for the Party and government. Currently, the government employs two primary approaches to environmental governance: routine governance and campaign-style governance (1).

Routine governance refers to enhancing the effectiveness of environmental management through the improvement of environmental laws and regulations, as well as the reinforcement of local governments’ primary responsibilities for environmental protection (2). Examples of this approach include institutional designs such as the ecological target responsibility system and the vertical management of environmental supervision. Campaign-style governance, on the other hand, involves mobilizing administrative resources with political support to accomplish specific environmental tasks within a set timeframe (3). Examples include central environmental inspections and environmental administrative talks. When routine governance fails due to issues such as principal-agent risks, conflicts between central and local government objectives, and weak performance incentives, the central government turns to campaign-style governance (1, 4).

The existing literature has extensively explored the effects of campaign-style governance, which can be categorized into three groups based on the targets of campaign-style enforcement. The first group focuses on the impact of campaign-style enforcement on local governments behavior and economic growth (5, 6). For example, Van Rooij (6), using case studies, found that while campaign-style enforcement can compel local governments to enforce regulations strictly in the short term, it fails to address the fundamental issue of lax enforcement by local governments. The second group of studies found that campaign-style enforcement can enhance public environmental awareness and stimulate public enthusiasm for participating in environmental protection (7–9). The third group of literature examines the impact of campaign-style enforcement on enterprises (3, 10). For instance, Liu et al. (3) used a case study approach to analyze the measures and effects adopted by power generation enterprises in response to campaign-style enforcement. In conclusion, although existing literature has examined the impact of campaign-style enforcement on corporate environmental governance, it has overlooked the moderating effect of government-business relations on the micro-level effectiveness of such enforcement.

The Central Environmental Inspection (CEI) is a nationwide environmental supervision program launched by the Chinese central government to strengthen the enforcement of environmental laws and regulations (7). CEI teams are led by recently retired provincial- or ministerial-level officials and are dispatched to provincial-level regions to assess how well local governments have implemented national environmental policies. During the inspection, CEI teams conduct field investigations and collect public complaints to identify environmental violations. After the inspection, each province is required to submit a rectification plan within a specified period, and the central government closely monitors its implementation.

The CEI represents a typical campaign-style enforcement mechanism: it is backed by strong political authority, features extensive public participation, and involves a strict accountability system for local officials (11). First piloted in Hebei Province in 2016, the CEI was subsequently expanded nationwide in several batches, as shown in Table 1. This staggered rollout provides an excellent quasi-experimental setting to examine how campaign-style environmental enforcement affects corporate environmental governance.

**Table 1:** Basic information of different batches of first CEI.

Inspection batch	Inspected provinces	Inspected time	Announcement time of rectification plan
Pilot	Hebei	2016.01–2016.02	2016.07
1st batch	Inner Mongolia, Heilongjiang, Jiangsu, Jiangxi, Henan, Guangxi, Yunnan, Ningxia	2016.07–2016.08	2017.04
2nd batch	Beijing, Shanghai, Hubei, Guangdong, Chongqing, Shaanxi, Gansu	2016.11–2016.12	2017.07
3rd batch	Shanxi, Anhui, Tianjin, Hunan, Fujian, Liaoning, Guizhou	2017.04–2017.05	2017.12
4th batch	Jilin, Zhejiang, Shandong, Hainan, Sichuan, Tibet, Qinghai, Xinjiang	2017.08–2017.09	2018.05

While originating as a campaign-style initiative, the CEI has gradually developed into an institutional arrangement aimed at strengthening vertical supervision and accountability between the central and local governments (12). It was designed not merely as a temporary campaign to rectify pollution problems but as a mechanism to strengthen the central government’s vertical supervision and accountability over local environmental enforcement. In China’s multi-level governance structure, local governments often face conflicting incentives between economic growth and environmental protection, leading to selective or lax enforcement of environmental regulations (13, 14). By introducing a centralized inspection and rectification mechanism, the CEI helps mitigate the principal–agent problem between the central and local governments, thereby bridging the gap between environmental policy formulation and implementation (11). In this sense, the institutionalization of the CEI has not only strengthened vertical supervision and accountability but also improved the overall coherence and effectiveness of China’s environmental governance system.

Building on this institutional background, this paper takes the CEI as a quasi-natural experiment to empirically examine how campaign-style enforcement affects corporate environmental governance and how its effectiveness is moderated by government–business relations. The study finds that campaign-style enforcement can prompt heavily polluting enterprises to increase their environmental investments, while the closeness of government-business relations negatively moderates this effect. Furthermore, under the pressure of the CEI, local governments mainly use punitive measures rather than financial subsidies to prompt heavily polluting enterprises to increase their environmental investments. Heterogeneity analysis reveals that campaign-style enforcement has a more pronounced effect on small firms and firms located in eastern regions.

Compared to existing literature, this paper makes three key contributions. First, the existing literature predominantly examines the effectiveness of campaign-style enforcement, while largely neglecting the moderating role of government-business relations on its outcomes (10, 15). This study supplements the existing literature by identifying micro-level variations in the effects of campaign-style enforcement across regions with differing levels of government-business relations. Second, previous research has demonstrated that the CEI enhances environmental performance at the macro level (7, 16). However, the micro-level mechanisms underlying this improvement remain unclear. This study contributes by providing firm-level evidence on how the CEI improves environmental performance. Third, compared to existing literature, this paper further explores the heterogeneity of the micro-level effects of campaign-style enforcement (e.g., firm size and firm location). These findings not only enrich the perspectives of current research but also provide significant practical implications for the implementation of future CEI.

## 2 Literature review

Campaign-style enforcement is an important method for bridging enforcement gaps (17). When routine governance fails, the government often resorts to campaign-style enforcement to achieve policy objectives (1). Compared to routine governance, campaign-style enforcement offers several advantages: it is implemented in a top-down manner with clear objectives, benefits from strong political backing, enables large-scale public mobilization, and is supported by strict accountability mechanisms (3, 18).

In China, to improve environmental quality in the short term, both the central and local governments widely adopt campaign-style enforcement. For example, to ensure that participants can enjoy blue skies during major events held in China, local governments in host cities and surrounding areas implement a series of pollution control measures, including halting industrial production, suspending construction activities, and restricting traffic (8).

Although campaign-style enforcement can achieve positive environmental outcomes by curbing corporate environmental violations (3, 7), it continues to face criticism. On one hand, campaign-style enforcement may hinder the establishment of regular enforcement mechanisms and undermine the rule of law (19); on the other hand, it may have negative impacts on local economic development and employment due to the shutdown of enterprises (20). Additionally, campaign-style enforcement may lead to public skepticism about the government’s administrative capabilities (1) and weaken citizens’ political support (21).

The effectiveness of campaign-style enforcement is a focal point of debate in the current literature. Some scholars argue that the effects of campaign-style enforcement are short-term because it fails to address fundamental conflicts of interest (6). However, recent studies suggest that effective campaign-style enforcement can raise public environmental awareness and participation, which may lead to long-term impacts (8, 9). The findings of Jia and Chen (7) also support the notion that campaign-style enforcement has long-term policy effects. Using the Central Environmental Inspection as an example, they explored the impact of campaign-style enforcement on environmental performance. However, their study did not analyze how the Central Environmental Inspection improves environmental performance, what the micro-level mechanisms are, or how it specifically affects corporate environmental governance.

Additionally, the existing literature generally agrees that local protectionism is a major obstacle to the effective implementation of environmental laws in China (22–25). When environmental enforcement has the potential to restrict local economic growth, government revenue, and employment, local governments often use their authority in environmental enforcement to protect local enterprises (26). Thus, the question arises: can campaign-style enforcement help mitigate or overcome these obstacles posed by local protectionism in environmental enforcement? Therefore, this paper uses the Central Environmental Inspection as an example to explore the impact of campaign-style enforcement on corporate environmental governance and further investigates the differences in the effects of campaign-style enforcement across regions with varying government-business relations.

## 3 Theoretical analysis and hypothesis

### 3.1 The impact of the campaign-style enforcement on corporate environmental governance

The CEI is a nationwide environmental initiative launched by the Chinese central government, characterized by top-down political mobilization, extensive public participation, and strict accountability—typical features of campaign-style enforcement (11). By linking inspection results to the performance evaluation and promotion of local officials (27, 28), the CEI reinforces the political salience of environmental protection and exerts strong pressure on local governments to act (17).

Under such political pressure, local governments often adopt intensive enforcement measures such as concentrated inspections, production restrictions, or the suspension and shutdown of operations to ensure compliance (8, 20). Heavily polluting firms, given their high emissions and environmental risks, are the main targets of these inspections and face stricter supervision and higher accountability pressure than other firms, while non-heavily polluting firms are subject to relatively lighter constraints. Although these rectification measures may cause short-term production losses, they also motivate heavily polluting firms to increase environmental investment by installing pollution-control equipment, improving production processes, or expanding green R&D to reduce the risk of penalties and accountability (29, 30).

In addition, the CEI includes a “look-back” mechanism that continuously monitors rectification progress and enforces accountability (31). This mechanism discourages temporary or symbolic compliance and encourages firms to adopt long-term strategies such as investing in green R&D and installing energy-saving and emission-reduction equipment. Based on the above analysis, the following hypothesis is proposed:

*H1*: Compared to non-heavily polluting firms, Central Environmental Inspections prompt heavily polluting enterprises to increase their environmental investments

### 3.2 The moderating effect of government-business relations on the policy impact of the central environmental inspection

In China’s environmental governance system, the relationship between the central and local governments can be viewed as a principal–agent structure (31). The central government sets environmental goals and evaluates performance, while local governments are responsible for implementation (32). However, due to conflicting objectives, local governments often prioritize economic growth over environmental protection, especially under the previous “GDP competition” model for official promotion (13, 14).

When local governments pursue short-term GDP growth and fiscal revenues, they may relax environmental enforcement to attract or protect local enterprises. These behaviors create opportunities for collusion between governments and firms (26, 33). Such collusion is a manifestation of the deterioration of government-business relations, leading to a divergence between the effectiveness of local environmental governance and the central government’s intended goals.

the closeness of government–business relations is expected to moderate the policy effects of the CEI. In regions with closer government–business ties, the intensified enforcement associated with the CEI is less likely to be sustained after the inspection cycle, weakening firms’ incentives to continue environmental investment. In contrast, where such ties are weaker, the impact of the CEI tends to be more persistent and firms are more likely to increase environmental investment. Based on this analysis, Hypothesis 2 is proposed:

*H2*: Government–business relations negatively moderate the impact of the CEI on firms’ environmental investment.

## 4 Research design

### 4.1 Sample data

This study uses annual data from A-share listed companies for the period from 2012 to 2019. Data before 2012 were not included primarily due to the limited disclosure of corporate environmental investment in annual reports during that time. Additionally, data from 2020 onwards were excluded to avoid potential biases caused by extreme market fluctuations related to the COVID-19 pandemic, which could significantly distort corporate environmental investment behavior.

The initial sample was processed as follows: firms subject to ST (Special Treatment) or PT (Particular Treatment) were excluded; financial and insurance companies were removed; observations with missing values were discarded, and firms with zero environmental investment throughout the entire study period were also excluded. Ultimately, the final sample consists of 3,170 firm-year observations.

The data on central environmental inspections and corporate environmental investments were manually collected by the authors. All other data were obtained from the China Stock Market & Accounting Research (CSMAR) database. To reduce the impact of outliers on the regression results, all continuous variables in the regression model were winsorized at the 1st and 99th percentile levels.

### 4.2 Empirical model

The difference-in-differences (DID) method is commonly used to estimate the effects of policy interventions (34). However, since the first round of central environmental inspections was implemented in different provinces in stages, it does not meet the requirement of the standard DID method for policy implementation at the same time point. Therefore, this study employs a staggered DID approach. Drawing on the research by Jia and Chen (7), we construct the following regression model:


(1) $$EI_{i,t}=\beta_{0}+\beta_{1}\mathrm{CE}I_{i,t}\times {\text{Pind}}_{i}+\lambda X_{i,t}+\delta_{i}+\eta_{t}+\varepsilon_{i,t}$$

In Equation 1, *EI_i,t_* represents the standardized environmental investment amount of firm *i* in year *t*, multiplied by 100. *CEI_i,t_* is a dummy variable indicating whether the province of firm *i* underwent central environmental inspections in year *t*. *Pind_i_* is a dummy variable indicating whether firm *i* is a heavily polluting enterprise. *δ_i_* and *η_t_* represent firm fixed effects and time fixed effects, respectively, while *ε_i,t_* denotes the random error term.

A critical prerequisite for applying the DID model for policy assessment is to satisfy the parallel trends assumption. This implies that the environmental investment trends of firms in the “treatment group” and “control group” should be similar before the CEI. To test this assumption and analyze the dynamic effects of the policy, we refer to related research (35, 36) and construct the following model.


(2) $$\begin{array}{c} EI_{i,t}=\beta_{0}+\sum_{t=-5}^{t=-1}\beta_{k}{\text{Before}}_{t}\times {\text{Pind}}_{i}+\sum_{t=0}^{t=3}\beta_{k}{\text{After}}_{t}\times {\text{Pind}}_{i}+\lambda X_{i,t}\\ +\delta_{i}+\eta_{t}+\varepsilon_{i,t} \end{array}$$

In Equation 2, the key explanatory variables are a series of dummy variables, *Beforeₜ* and *Afterₜ*, which capture the differences in environmental investment between the treatment and control groups in each year before and after the CEI. Specifically, *Beforeₜ* (*Afterₜ*) equals 1 if the observation occurs t years before (after) the arrival of the CEI team in a province, and 0 otherwise. The year immediately preceding the CEI (t = −1) serves as the benchmark period. The coefficients of Beforeₜ are used to verify whether firms in the treatment and control groups follow similar pre-policy trends (i.e., the parallel trends assumption), while those of Afterₜ reflect the dynamic effects of the CEI over time. The definitions of all other variables remain consistent with those in Equation 1.

### 4.3 Variables definition

#### 4.3.1 Explained variables

Drawing on the studies by Zhang et al. (37) and Zhong et al. (38), we manually collected data on corporate environmental investment. This data refers to the total expenditure directly related to environmental projects, such as wastewater treatment, desulfurization projects, and waste gas management, which are detailed under the “Construction in Progress” section of listed companies’ annual reports. To account for differences in company size, we standardized the corporate environmental investment by dividing it by the company’s total assets at the end of the year. Additionally, to enhance the readability of the regression coefficients, the standardized corporate environmental investment is multiplied by 100.

#### 4.3.2 Explanatory variable

CEI indicates whether the province where a listed company is registered has undergone central environmental inspection. When the central environmental inspection team is stationed in and conducts inspections in a particular province, the CEI value for listed companies in that province is set to 1, while for other provinces, it is set to 0. Considering that CEI involves continuous monitoring of local governments’ rectification efforts through a “look-back” mechanism, this study assigns a CEI value of 1 to listed companies in provinces even after the inspections have concluded.

Pind represents a dummy variable for heavily polluting enterprises. It is determined based on the “Industry Classification and Management Directory for Environmental Protection Verification of Listed Companies” issued by the former Ministry of Environmental Protection in 2008, in conjunction with the “Guidelines for the Industry Classification of Listed Companies” revised by the China Securities Regulatory Commission in 2012. If a company is classified as a heavily polluting enterprise according to these criteria, Pind is set to 1; otherwise, it is set to 0.

#### 4.3.3 Moderating variable

Due to the lack of a widely accepted and directly measurable indicator for government-business collusion behavior in existing research, this study adopts the “Close Index” from the “Ranking of Government–Business Relations in Chinese Cities (2017, Full Data Edition)” published by Nie et al. (39) as a proxy for government-business collusion, denoted as GBR. A higher value of GBR indicates a closer government-business relations.

#### 4.3.4 Control variables

Drawing on related research (37, 38), this study selects several control variables, including firm size (Size), financial leverage (Lev), profitability (Roe), firm growth (Growth), and agency costs (AgencyCost), among others. Detailed definitions of these variables are provided in Table 2.

**Table 2:** Variable definition.

Symbol	Variable description
EI	Environmental investment *100/ Total assets
CEI	When the province where the listed company is located undergoes CEI, CEI is equal to 1. It remains 1 even after the inspection ends. Otherwise, it takes the value of 0
Pind	When the company is in the heavy pollution industry, Pind is equal to 1, and 0 otherwise
GBR	GBR is the close index of the “Ranking of Political and Business Relations in Chinese Cities (2017)”
Size	The natural logarithm of total assets
Lev	Total liabilities/total assets
Growth	Operating income growth/Total operating income of the previous year
Roe	Net profit/Average shareholders’ equity
Soe	Takes a value of 1 when state-owned, and 0 otherwise
Power	When the chairman concurrently serving as CEO, Power is equal to 1, and 0 otherwise
Inst	Shareholding of institutional investors
Top1	Shareholding of major shareholders
AgencyCost	Administration expense/Operating income

## 5 Empirical results

### 5.1 Descriptive statistics

The descriptive statistics for all variables are presented in Table 3. The average value of EI is 0.222%, with a standard deviation of 0.748, a minimum value of 0, and a maximum value of 11.964%. This indicates that, even after standardization by total assets, there are still significant differences in environmental investments among firms, providing scope for further examination. The mean value of CEI is 0.447, suggesting that about 45% of firm-year observations experienced central environmental inspections. The mean value of Pind is 0.489, indicating that approximately half of the firm-year observations in the sample belong to heavily polluting industries. The GBR variable ranges from a minimum of 2.472 to a maximum of 100, showing substantial variation in the proximity index of government-business relations across different cities. For the control variables, the statistical values of Size, Lev, Growth, Roe, and others are generally consistent with the findings of existing studies.

**Table 3:** Descriptive statistics.

Variable	*N*	Mean	S. D.	Min	P50	Max
EI	3,170	0.222	0.748	0.000	0.000	11.964
CEI	3,170	0.447	0.497	0.000	0.000	1.000
Pind	3,170	0.489	0.500	0.000	0.000	1.000
GBR	3,170	48.744	23.105	2.472	45.342	100.000
Size	3,170	22.973	1.436	19.585	22.818	28.341
Lev	3,170	0.466	0.197	0.014	0.469	1.118
Growth	3,170	0.204	1.583	−0.862	0.091	56.174
Roe	3,170	0.066	0.334	−14.706	0.077	1.332
Soe	3,170	0.529	0.499	0.000	1.000	1.000
Power	3,170	0.216	0.412	0.000	0.000	1.000
Inst	3,170	38.716	23.280	0.000	41.025	92.704
Top1	3,170	37.056	15.452	3.622	35.679	89.986
Agencycost	3,170	19.593	1.319	16.262	19.458	25.168

### 5.2 Parallel trends test and dynamic effects

**Figure 1:**
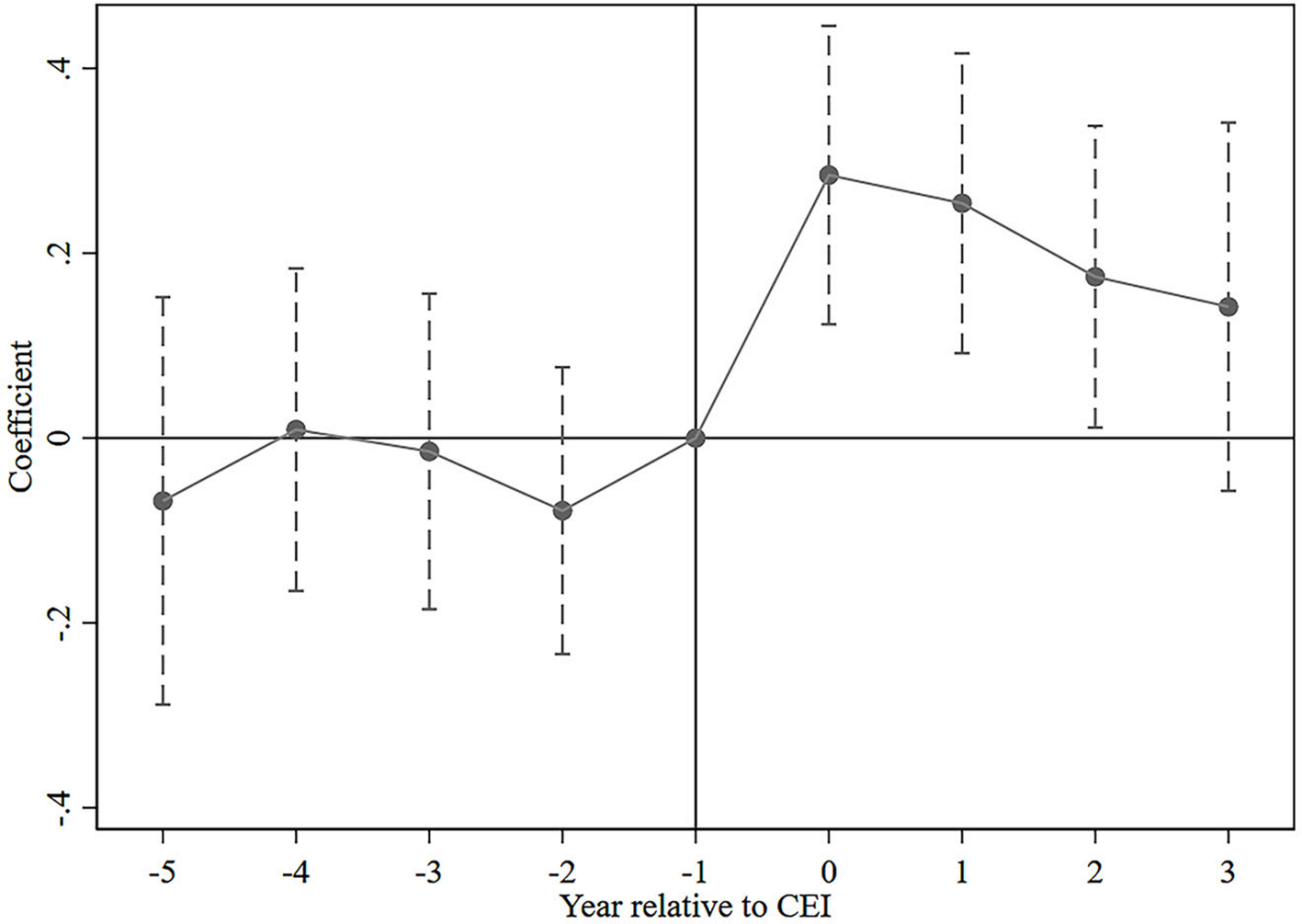
Parallel trends test.

To visualize the regression results, we present the trend of the regression coefficients for *Before_t_* and *After_t_* in Figure 1, with dashed lines representing the 95% confidence intervals. As shown in Figure 1, when t < −1, the coefficients for *Before_t_* are not significant, indicating no significant differences in environmental investment trends between the treatment and control groups before the CEI. Therefore, the parallel trend assumption is satisfied. When t > −1, the *After_t_* coefficients are significantly positive and show a decreasing trend, although the coefficient for *After_3_* is not significant. This suggests that the CEI significantly increased corporate environmental investment with some lasting effects, though these effects gradually weakened over time. This finding is consistent with the characteristics of campaign-style enforcement, which tends to lack sustained impact due to unresolved conflicts of interest between central and local governments (6).

### 5.3 Baseline results: the effect of the CEI on corporate environmental investments

Table 4 presents the regression results on the impact of central environmental inspections on corporate environmental investments. In the full sample regression, the coefficient of CEI × Pind is significantly positive at the 1% confidence level, indicating that CEI significantly prompts heavily polluting firms to increase their environmental investments. This suggests that campaign-style enforcement can promote environmental management among firms, thus supporting Hypothesis 1. This empirical result provides firm-level evidence for the effectiveness of campaign-style enforcement in improving environmental performance and serves as an important supplement to the studies by Jia and Chen (7) and Feng et al. (16).

**Table 4:** the effect of CEI on corporate environmental investments.

Variables	Full sample	Pilot	First batch	Second batch	Third batch	Fourth batch
	EI	EI	EI	EI	EI	EI
CEI × Pind	0.158*** (0.043)	−0.690** (0.274)	0.315*** (0.115)	0.161** (0.065)	0.245** (0.115)	0.233 (0.164)
Control variables	Yes	Yes	Yes	Yes	Yes	Yes
Firm FE	Yes	Yes	Yes	Yes	Yes	Yes
Year FE	Yes	Yes	Yes	Yes	Yes	Yes
Constant	1.083 (0.702)	6.253 (4.549)	1.463 (2.836)	1.534 (1.303)	0.128 (1.806)	0.307 (1.470)
*N*	3,170	57	603	1,060	593	830
*R* ^2^	0.423	0.648	0.443	0.304	0.506	0.425

Robust standard errors are reported in parentheses. ***p < 0.01, **p < 0.05, *p < 0.1.

Furthermore, since the CEI was gradually extended from pilot provinces to nationwide implementation, firms in different batches exhibited heterogeneous responses. In the pilot batch, corporate environmental investment declined significantly, likely because enforcement was particularly stringent, with heavy penalties and even production suspensions, which raised short-term compliance costs and crowded out investment. In addition, pilot regions typically faced greater environmental pressure and long-standing problems, where firms had already invested substantially, leaving limited room for further increases. As the CEI became institutionalized and policy expectations stabilized, corporate environmental investment rose significantly in subsequent batches.

In the batch-by-batch regressions (see Table 4), the coefficients of CEI × Pind remain significantly positive in the first to third batches, although the level of significance declines; by the fourth batch, the coefficient is no longer significant. This suggests that heavily polluting firms in later rounds indeed learned from earlier inspections, indicating a “learning effect,” although this effect weakened over time.

### 5.4 The moderating effect of government-business relations on the effectiveness of the CEI

Table 5 reports the regression results for the moderating effect of government–business relations on the effectiveness of the CEI. Columns (1), (2), and (3) present different model specifications: model (1) includes only control variables, model (2) further adds year-fixed effects, and model (3) additionally controls for both year and firm-fixed effects. The coefficients of the interaction term *CEI × Pind × GBR* are consistently negative and statistically significant across all models, indicating that in regions with closer government–business relations, the CEI’s effect on promoting corporate environmental investment tends to be weaker. This empirical evidence, based on a large sample, is consistent with the conclusion of Van Rooij (6), who argues that while campaign-style enforcement can temporarily enhance environmental performance, it often fails to resolve the underlying issue of local protectionism.

**Table 5:** The moderating effect of government-business relations on the effectiveness of the CEI.

Variables	(1)	(2)	(3)
	EI	EI	EI
CEI × Pind	0.471*** (0.097)	0.426*** (0.095)	0.268*** (0.093)
GBR	−0.001*** (0.000)	−0.001*** (0.000)	0.000 (0.000)
CEI × Pind × GBR	−0.004** (0.002)	−0.004** (0.002)	−0.002* (0.002)
Control variables	Yes	Yes	Yes
Firm FE	No	No	Yes
Year FE	No	Yes	Yes
Constant	1.124*** (0.250)	1.136*** (0.245)	1.053 (0.696)
*N*	3,170	3,170	3,170
*R* ^2^	0.048	0.063	0.424

Robust standard errors are reported in parentheses. ***p < 0.01, **p < 0.05, *p < 0.1.

### 5.5 Robust test

#### 5.5.1 Placebo test

The regression results in this study could be influenced by other random factors. To test the robustness of our conclusions, we conducted a placebo test. Specifically, following the approach of Topalova (40), we set the sample period from 2012 to 2015 and advanced the timing of the CEI by 2 years, after which we conducted the regression analysis. The regression results are presented in Columns (1) and (2) of Table 6. The findings show that the coefficients of CEI × Pind and CEI × Pind ×GBR are not statistically significant, indicating that the hypothetical CEI does not have a significant impact on environmental investment by firms in heavily polluting industries. Furthermore, the moderating effect of government-business relations on the effectiveness of central environmental inspections is also not significant. Therefore, the conclusions of our study are reliable.

**Table 6:** Robust test.

Variables	Placebo test		PSM-DID		Control environment tax	
	(1)	(2)	(3)	(4)	(5)	(6)
	EI	EI	EI	EI	EI	EI
CEI × Pind	−0.041 (0.048)	−0.044 (0.111)	0.256*** (0.043)	0.370*** (0.079)	0.159*** (0.044)	0.269*** (0.094)
GBR		0.000 (0.000)		0.000 (0.000)		0.000 (0.000)
CEI × Pind ×GBR		0.000 (0.002)		−0.003* (0.001)		−0.003* (0.002)
Env_tax ×Post					−0.006 (0.053)	−0.011 (0.054)
Control variables	Yes	Yes	Yes	Yes	Yes	Yes
Firm FE	Yes	Yes	Yes	Yes	Yes	Yes
Year FE	Yes	Yes	Yes	Yes	Yes	Yes
Constant	1.220 (1.012)	1.224 (1.007)	0.795 (0.733)	0.746 (0.855)	1.084 (0.703)	1.055 (0.696)
*N*	1,406	1,406	2,834	2,834	3,170	3,170
*R* ^2^	0.472	0.472	0.422	0.423	0.423	0.424

Robust standard errors are reported in parentheses. ***p < 0.01, **p < 0.05, *p < 0.1.

#### 5.5.2 Use propensity score matching (PSM) plus DID

To mitigate estimation bias due to sample selection issues, we further employed the PSM-DID approach for robustness testing. Specifically, this study utilizes firm data before the event (i.e., data from 2012 to 2015) and employs the radius matching method (with a radius of 0.05) to match the treatment and control group samples. Subsequently, the matched samples are used to examine the impact of the CEI on corporate environmental investment and the moderating effect of government-business relations. In column (3) of Table 6, the coefficient of CEI × Pind is significantly positive at the 1% confidence level; in column (4) of Table 6, the coefficient of CEI × Pind ×GBR is significantly negative at the 10% confidence level. This suggests that the CEI effectively promoted increased environmental investment among heavily polluting enterprises, but the government-business relations negatively moderated the effect of the CEI. These findings are consistent with our earlier results, indicating that the conclusions of our study are reliable.

#### 5.5.3 Control for the environmental protection tax law

To further test the robustness of our findings and to eliminate potential confounding effects from concurrent environmental policies, we control for the possible impact of the Environmental Protection Tax Law, which was officially implemented in 2018. This policy requires polluting enterprises to pay taxes based on the volume of their pollutant emissions, thereby potentially influencing corporate environmental behavior through the mechanism of “increased pollution costs leading to greater environmental investment.” Following the approach of Jin et al. (41), we construct an interaction term (Env_tax ×Post) by multiplying firms located in regions with higher increases in environmental tax burdens by the post-implementation period of the law. We then incorporate this interaction into the baseline model to identify and control for the potential confounding effects of the Environmental Protection Tax Law on our estimates.

As reported in Column (5) of Table 6, the coefficient on CEI × Pind remains significantly positive at the 1% level. In Column (6), the coefficient on CEI × Pind ×GBR is significantly negative at the 10% level. These results indicate that, even after controlling for the effects of the environmental tax policy, the Central Environmental Inspection (CEI) continues to significantly promote corporate environmental investment, while closer government–business relations negatively moderate the effect of the CEI. Overall, these findings confirm that the main conclusions of this paper are not driven by the contemporaneous environmental tax policy but rather reflect the independent governance effect of the CEI.

### 5.6 Mechanism test: punitive mechanism or incentive mechanism?

The research findings suggest that the CEI contributes to encouraging heavily polluting enterprises to increase their environmental investments. However, the specific mechanisms through which the CEI influences corporate behavior remain unclear, necessitating further exploration. The CEI exerts pressure on local governments to enforce central environmental policies, which in turn affect the environmental governance practices of enterprises. In this context, local governments typically adopt one or both of the following measures to achieve environmental governance objectives: imposing environmental penalties on enterprises or providing environmental subsidies.

Environmental penalties result in direct economic losses for companies and have a negative impact on their stock value (42), causing the financial losses from environmental violations to exceed their environmental governance costs. Therefore, based on the cost–benefit principle, companies will increase their environmental investments to reduce the risk of environmental violations (43). Compared to environmental penalties, government environmental subsidies represent a form of positive incentive. Faced with the dual pressures of economic growth and environmental protection, local governments may opt to provide environmental subsidies to support enterprises in making environmental investments, helping them meet the rectification tasks assigned by CEI on time. Environmental subsidies not only reduce the cost of environmental management for enterprises but also improve their cash flow and overall performance. As a result, government environmental subsidies can help bridge the funding gap in environmental governance and promote corporate environmental investment.

Therefore, to examine the specific mechanisms through which the Central Environmental Inspection affects corporate environmental governance, we construct the following model:


(3) $$\mathrm{Fin}e_{i,t}/\text{Subsid}y_{i,t}=\beta_{0}+\beta_{1}\mathrm{CE}I_{i,t}\times {\text{Pind}}_{i}+\lambda X_{i,t}+\eta_{t}+\mu_{i}+\varepsilon_{i,t}\;$$

In Equation 3, *Fine_i,t_* represents the natural logarithm of the amount of environmental fines imposed on the enterprise, and *Subsidy_i,t_* represents the amount of environmental subsidies standardized by total assets. *X* denotes a set of control variables. Based on existing literature, we select Size, Lev, Roe, and Growth as control variables. Other variables remain consistent with Model (1). The data on the amount of environmental fines comes from the China Research Data Service Platform (CRNDS), while the data on environmental subsidies is extracted from the notes in the companies’ financial reports and collected manually.

The regression results are presented in Table 7. In column (1), the regression coefficient of CEI × Pind is 0.214, which is positively significant at the 10% confidence level. This indicates that the CEI indeed motivates local governments to intensify environmental penalties. In column (2), the regression coefficient of CEI × Pind is −0.001, which is not significant, suggesting that the CEI does not incentivize local governments to increase environmental subsidies. Therefore, the CEI primarily promotes increased environmental investment by heavily polluting enterprises through punitive mechanisms rather than through incentive mechanisms.

**Table 7:** Mechanism test.

Variables	(1)	(2)
	Fine	Subsidy
CEI × Pind	0.214* (0.111)	−0.001 (0.003)
Control variables	Yes	Yes
Firm FE	Yes	Yes
Year FE	Yes	Yes
Constant	1.191 (2.652)	−0.040 (0.051)
*N*	3,170	3,170
*R* ^2^	0.269	0.490

Robust standard errors are reported in parentheses. ***p < 0.01, **p < 0.05, *p < 0.1.

### 5.7 Heterogeneity analysis

#### 5.7.1 The impact of firm scale

Due to the importance of large enterprises in local economic development, taxation, and employment, local officials may assist these enterprises in circumventing environmental regulations under the pressure of performance evaluations (44), resulting in government-business collusion. Based on the median firm size in 2015, the sample was divided into large and small enterprises and regression analyses were conducted separately for each group. The regression results are presented in Columns (1) and (2) of Table 8. The results show that for large enterprises, the coefficient of CEI × Pind is not significant, while for small enterprises, the coefficient is significantly positive at the 5% confidence level. This indicates that the CEI significantly increased environmental investment for small enterprises, but had no significant effect on large enterprises. These findings further support Hypothesis 2, which posits that local governments are more likely to collude with large enterprises, thereby weakening the effect of the CEI, compared to small enterprises.

**Table 8:** Heterogeneity analysis.

Variables	Sub sample: firm scale		Sub sample: region		
	Large	Small	Eastern	Central	Western
	EI	EI	EI	EI	EI
CEI × Pind	0.103 (0.072)	0.143** (0.068)	0.151*** (0.050)	0.201* (0.112)	0.121 (0.155)
Control variables	Yes	Yes	Yes	Yes	Yes
Firm FE	Yes	Yes	Yes	Yes	Yes
Year FE	Yes	Yes	Yes	Yes	Yes
Constant	1.781 (2.124)	3.520*** (1.197)	1.842** (0.741)	−0.863 (1.869)	2.889 (2.150)
*N*	1,444	1,692	2,168	580	422
*R* ^2^	0.313	0.541	0.475	0.445	0.333

Robust standard errors are reported in parentheses. ***p < 0.01, **p < 0.05, *p < 0.1.

#### 5.7.2 The impact of region

The level of regional economic development significantly influences corporate environmental governance (38). Accordingly, this study categorizes the sample into three groups—eastern, central, and western regions—based on the registered addresses of listed companies to analyze regional differences. Separate regressions are conducted for each group. Columns (3) to (5) of Table 8 present the regression results for the regional heterogeneity of the CEI. The results show that the coefficient of CEI × Pind is significantly positive at the 1% confidence level in the eastern region and at the 10% confidence level in the central region, while it is statistically insignificant in the western region. This indicates that the CEI significantly promoted environmental investments among firms in the eastern and central regions, whereas its impact on firms in the western region was not significant.

These results suggest that the CEI exerts a stronger governance effect in regions with higher levels of economic development. In the eastern region, where market institutions are more mature and fiscal capacity is stronger, local governments face less economic pressure and have greater administrative resources to implement environmental rectification measures (45). Consequently, the CEI significantly enhances firms’ environmental investments in this region. In the central region, the CEI also shows a positive but weaker effect, which may reflect a gradual improvement in regulatory enforcement as local governments balance economic growth and environmental protection. In contrast, the coefficient in the western region is insignificant, likely due to higher economic and fiscal pressures that constrain local governments’ enforcement capacity (46). Under such conditions, local officials may prioritize economic performance or develop collusive relationships with enterprises to sustain growth (6, 33).

Overall, the regional heterogeneity results in Table 8 provide partial support for Hypothesis 2, indicating that local governments facing greater economic pressure are more prone to collusion with enterprises, thereby weakening the effectiveness of environmental policies.

## 6 Conclusions and implications

Campaign-style enforcement is a significant approach employed by the Chinese government to achieve environmental governance (3, 7). While existing literature has examined the impact of this enforcement on corporate environmental governance, it has overlooked the moderating effect of government-business relations on the micro-level effectiveness of such enforcement. This paper uses the example of the CEI to examine the impact of campaign-style enforcement on corporate environmental governance and its underlying mechanisms. It further investigates the moderating role of government-business relations on the effectiveness of campaign-style enforcement. The study finds that campaign-style enforcement can prompt heavily polluting enterprises to increase environmental investment, while the closeness of government-business relations negatively moderates the effectiveness of such enforcement. Furthermore, under the pressure of the CEI, local governments primarily employ punitive measures rather than financial incentives to prompt these enterprises to increase their environmental commitments. Heterogeneity analysis reveals that campaign-style enforcement has a more pronounced effect on small firms and firms located in eastern regions.

Based on the above findings, this study offers the following policy implications:

First, the existing performance evaluation system for officials should be optimized. The study finds that collusion between government and enterprises weakens the effectiveness of campaign-style enforcement, highlighting deficiencies in the current evaluation mechanism. Therefore, this paper suggests increasing the emphasis on environmental performance in the evaluation system, linking environmental goals to the promotion and assessment of local officials. This approach aims to reduce the motivation for local officials to sacrifice environmental protection for political gains and to curb the collusion between local governments and enterprises.

Second, supervision of large enterprises should be strengthened. The study finds that Central Environmental Inspections have had no significant impact on the environmental investments of large enterprises, possibly due to collusion with local governments. Thus, The Central Environmental Inspection Team should strengthen supervision of large enterprises to prevent them from evading environmental regulations through rent-seeking and interest exchange. Measures such as increasing penalties for environmental violations by large enterprises and enhancing public exposure of such violations can serve as stronger deterrents.

Third, financial support and technical assistance to undeveloped regions should be increased. In economically underdeveloped areas, such as the central and western regions, local governments may be more inclined to relax environmental requirements to support local economic development. Therefore, the central government should reduce the reliance of underdeveloped regions on traditional economic growth models by increasing fiscal support, technical assistance, and other targeted measures. Specific policies could include establishing special funds to promote the development of green industries in these areas or offering tailored technical training to enhance the environmental protection capabilities of local enterprises. These actions would strengthen the capacity and willingness of these regions to pursue green development.

Undoubtedly, this study has several limitations. Only a subset of Chinese listed firms disclose detailed information on their annual environmental investments, which constrains the sample size and may affect the representativeness of the findings. In addition, due to the absence of a universally recognized or directly observable measure of government–business collusion, this study employs the close index from the Ranking of Government–Business Relations in Chinese Cities (2017, Full Data Edition) as a proxy variable. Although this indicator provides a reasonable approximation based on publicly available data, it may not fully reflect the multidimensional nature of government–business interactions. Future research could explore or construct more comprehensive and time-varying indicators to improve the robustness and generalizability of the findings.

## Data Availability

The raw data supporting the conclusions of this article will be made available by the authors, without undue reservation.
